# Emergency nurses’ perceptions regarding the risks appraisal of the threat of the emerging infectious disease situation in emergency departments

**DOI:** 10.1080/17482631.2020.1718468

**Published:** 2020-01-24

**Authors:** Stanley Kam Ki Lam, Enid Wai Yung Kwong, Maria Shuk Yu Hung, Wai Tong Chien

**Affiliations:** a School of Nursing, Tung Wah College, Kowloon, Hong Kong; b School of Nursing, Hong Kong Polytechnic University, Kowloon, Hong Kong; c Nethersole School of Nursing, The Chinese University of Hong Kong, New Territories, Hong Kong

**Keywords:** Emerging infectious diseases, Epidemics, qualitative research, emergency nurses, risk perception

## Abstract

**Purpose:** Emerging infectious diseases are considered as a pressing challenge to global public health. Throughout public health response to emerging infectious diseases, emergency nurses are situated at the forefront of the healthcare system. The present study has explored emergency nurses’ perceptions regarding the risks appraisal of the threat of the emerging infectious disease situation in emergency department context.

**Methods:** The present study used a qualitative descriptive approach. A purposive sampling method was employed to recruit emergency nurses who worked in public hospitals in Hong Kong. Semi-structured interviews were conducted to 24 emergency nurses. The data were interpreted using a thematic analysis strategy.

**Results:** Five overarching themes emerged from the data: (1) the novelty of an emerging infectious disease, (2) the severity of an emerging infectious disease, (3) the proximity to an emerging infectious disease, (4) the complexity of an emerging infectious disease situation, and (5) the response levels towards an emerging infectious disease situation.

**Conclusion:** It is anticipated that the information may help to predict the attitudes and behaviours of emergency nurses in future impending epidemic events, enhancing emergency nurses’ preparedness towards in such situations.

**Abbreviations:** EID: Emerging infectious disease; ED: Emergency department; SARS: Severe acute respiratory syndrome; MERS: Middle East respiratory syndrome; WHO: World Health Organization; RN: Registered nurse; APN: Advanced practice nurse; NO: Nursing officer

## Background

Emerging infectious diseases (EIDs) refer to communicable diseases that show an upward trend in incidence and prevalence in humans in the recent past or foreseeable future, in excess of the usual frequency in a given area or among a specified group of people over a particular period. These diseases could originate from both newly recognized and re-emerging existing infectious agents that present with increasing frequency and geographic range (Jones et al., 2008). EIDs often have enormous pandemic potential because of the minimal immunity among the human population, constituting one of the most pressing challenges to global public health (Frost, Li, Moolenaar, & Xie, 2019).

In the face of EIDs, emergency nurses are situated in the forefront of the healthcare system in combating EID outbreaks. Working at the emergency departments (EDs), emergency nurses act as the gatekeeper of the healthcare system by identifying suspected and confirmed patients with EID infections through meticulous assessment of reporting criteria such as disease manifestations, contact history, and travel history (Lam, Kwong, Hung, Pang, & Chien, 2019a). Also, the emergency nurses’ role in controlling the spread of EIDs by performing and maintaining high-quality infection control measures is imperative (Lam, Kwong, Hung, & Pang, 2016). Moreover, they are often involved in patient logistic arrangements within departments and hospitals. This task involves offering appropriate quarantine arrangements for suspected and confirmed patients in ED and allocating potential infectious patients to appropriate destinations and specialities (Lam & Hung, 2013). Such participations of emergency nurses in the management of EIDs are considered of vital importance for minimizing the risk of disease transmission among patients, staff, visitors, and communities (Lam, Kwong, Hung, Pang, & Chien, 2019b).

Despite the board involvement of emergency nurses in response to EIDs, their practices and performances are oftentimes reported to be inconsistent in the research to date. Previous studies on emergency nurses’ compliance with infection control recommendations and guidelines have highlighted that their performance could be seriously hampered during epidemics (Carter, Pouch, & Larson, 2014). For instance, in an observational study of emergency nurses’ adherence to infection prevention practices during the outbreak of Middle East respiratory syndrome (MERS) (Chacko & Camacho, 2016), it was revealed that more than 40% of the emergency nurses were not complying with the guidelines in the use of personal protective equipment. Also, although considered to be the single most important measure in the prevention of the spread of infections, almost one-fourth of the nurses in the study (23%) had demonstrated hand hygiene practices that fell below standard. Given that such inconsistencies of practice standard among emergency nurses would inevitably affect the public health preparedness and response capacity of healthcare systems, the need to understand the antecedents of the variations in their behaviours while performing duties against EIDs is highlighted.

To address the antecedents of emergency nurses’ attitudes and behaviours in EID management, understanding the components that influence their risk perception towards different EIDs is considered a useful approach. Individuals’ risk appraisal and perception, which depicts the perceived susceptibility to a threat to health, is considered the essential constituent that mediates attitude change and navigates decision making (Rogers, 1975; Slovic, 1987). This concept has been used extensively in the literature in understanding individuals’ judgement and motivation in certain health-related behaviours, such as treatment adherence (Garavalia, Garavalia, Spertus, & Decker, 2009; Kartal & Ozsoy, 2007) and vaccination (Raftopoulos, 2007; Rhodes & Hergenrather, 2003). The concept of risk perception has also been applied to the prediction of intentions and behaviours regarding the compliance of practice among health workers (Efstathiou, Papastavrou, Raftopoulos, & Merkouris, 2011). Indeed, previous research has established that nurses’ compliance with precautionary measures is primarily related to their personal anticipation of the level of risk for acquiring an infectious disease (Grady, Shortridge, Davis, & Klinger, 1993; Osborne, 2003; Williams, Campbell, Henry, & Collier, 1994). Taken together, these studies support the notion that understanding the factors influencing emergency nurses’ risk appraisal regarding different EIDs could offer relevant insights into their inconsistent attitudes and behaviours in EID management.

However, a significant knowledge gap exists in the understanding of emergency nurses’ perceptions regarding the risk appraisal of the threat of epidemic events. Although extensive research has been carried out in exploring the association between risk perception and decision-making among nurses and health workers in susceptible situations, previous studies have predominantly focused on the effects of risk being perceived on precautionary behaviours in infection control. There is a current paucity of empirical research focusing especially on the nature of an EID situation that influences risk appraisal. In addition, whilst some research has been carried out on the factors affecting nurses’ infection control practices, there have been few investigations into such area, particularly within emergency care settings. Thus, the present study aimed at addressing such knowledge gaps by exploring emergency nurses’ perceptions regarding the risks appraisal of the threat of the EID situation in the emergency department context. It is anticipated that the findings can provide useful information in understanding how emergency nurses appraise the risk from an epidemic event, addressing the existing knowledge gap regarding the inconsistencies of emergency nurses’ practices during different episodes of epidemics. Consequently, this might aid the development of strategies and tactic in enhancing the compliance and consistency among emergency nurses regarding the adoption of infection control measures during infectious disease outbreaks.

## Methods

### Design

The present study is part of a PhD project regarding the understanding of how emergency nurses are involved in the management of epidemic events. The larger project has generated publications about how emergency nurses perceive their roles and responsibilities during an epidemic event (Lam et al., 2019a) and the contextual factors influencing the practice of emergency nurses in managing emerging infectious diseases (Lam et al., 2019b).

A qualitative descriptive approach was adopted in the present study to explore the characteristics of an EID situation that would be influential to emergency nurses’ risk perception. The qualitative descriptive approach is a research method which aims at exploring the experiences, perceptions, and inclinations of individuals within a particular phenomenon (Kim, Sefcik, & Bradway, 2017). Employing a qualitative descriptive design can facilitate the understanding of the phenomenon of interest by gathering a rich and literal description of the insider views and perspectives (Bradshaw, Atkinson, & Doody, 2017). Considering that the present study aims at exploring the nature and characteristics of an EID situation that can affect emergency nurses’ risk perception from their experience, the qualitative descriptive design is used to generate a comprehensive summary that depicts and elicits these constituents in an explicit manner.

### Settings and participants

The present study was conducted in an emergency care setting, with the practice environment confined to the emergency departments (EDs) in regional acute care public hospitals in Hong Kong. A purposive sampling method was employed in the present study to recruit eligible participants. This sampling method can help researchers to facilitate the collection of relevant and useful information required for the study by identifying information-rich participants (Etikan, Musa, & Alkassim, 2016). With the use of a purposive sampling method, participants were recruited on the basis of their eligibility. Participants were considered eligible and invited to take part in the study if they (1) worked as a fulltime emergency nurse in public hospitals in Hong Kong; (2) were involved in active and direct provision of emergency care to patients at work, and (3) were willing to share their opinions and thoughts. Since the present study emphasized the experience of frontline emergency nurses, emergency nurses who were at the managerial position were excluded in the present study. Eligible individuals were approached through the social network of the members of the research team. Individuals who were interested in participating in the study were further contact through email. They were provided with the information sheet regarding the details of the study and the nature of their participation via email. The individuals were encouraged to ask questions for further information regarding the study. Participants who agreed to take part in the study were asked to sign an informed consent form on the date of data collection. Eventually, 24 emergency nurses participated in the present study. A summary of the demographic data of the participants was presented in Table I.

**Table I.** Demographic characteristics of the participants

Participant characteristics	Frequency (Percentage)n (%)
Age range (years)	
20–25	4 (16.7)
26–30	7 (29.2)
31–35	5 (20.8)
36–40	6 (25.0)
Above 41	2 (8.3)
Ranking	
Registered nurse	20 (83.3)
Advanced practice nurse	2 (8.3)
Nursing officer	2 (8.3)
Years of nursing experience	
1–5	7 (29.2)
6–10	9 (37.5)
11–15	6 (25.0)
16–20	2 (8.3)
Years in emergency departments	
1–5	14 (58.3)
6–10	3 (12.5)
11–15	6 (25.0)
16–20	1 (4.2)

### Data collection

**Figure 1.**
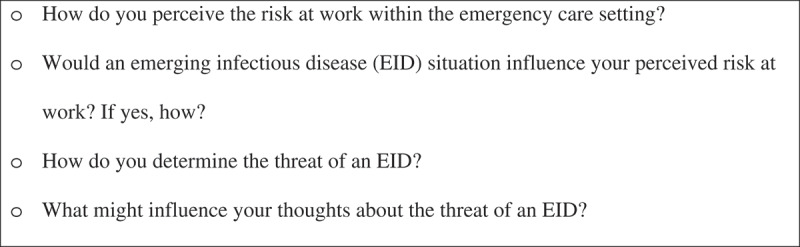
The interview guide

Semi-structured, face-to-face, individual interviews were conducted by the first author (SKKL) to solicit participants’ perceptions regarding the factors that were influential to the appraisal of the threat of an EID event. Interviews were arranged in quiet locations that were convenient to the participants, such as discussion rooms in community centres and public libraries. To facilitate the process of data analysis, all the interviews were audio-taped with the permission of the participants. Before the commencement of the interviews, participants were encouraged to freely express their views and opinions about the research area. An interview guide, which consists of open-ended questions, was used in the interview process (Figure 1). The interview guide could offer directions for the interviews while preserving flexibility for the participants to communicate their thoughts and ideas (McIntosh & Morse, 2015). The average time of the interviews was 90 minutes, with a range from 60 minutes to 120 minutes.

### Data analysis

Before data analysis, the audio-recorded interviews were transcribed verbatim on the same day right after each interview was completed. All the transcripts were checked against to make sure the correctness and accuracy of the transcription. Each transcript was then being analysed within one week following the completion of the corresponding interview. The process of data analysis in the present study followed the guideline of thematic analysis by Braun and Clarke (Braun & Clarke, 2006). As indicated in the guideline, the thematic analysis strategy consists of six primary phases: (1) familiarizing with data; (2) developing initial codes; (3) identifying possible themes; (4) refining preliminary themes; (5) finalizing relevant themes, and (6) producing a scholarly report. At the beginning of the data analysis process, the research team read and re-read the interview transcripts in a line-by-line and paragraph-by-paragraph manner to familiarize with the content of the data. Throughout the process, ideas from participants’ narratives that were considered relevant to the phenomenon under study were highlighted to develop an initial list of codes. After scrutinizing the transcripts, codes with related meanings or consistent patterns were sorted to form themes. Themes were also generated through collating coherent codes across the entire dataset. These themes were examined again for the refinement of the structures within and between themes, developing a network of clustered and revised themes and sub-themes. Themes that were considered relevant and important to the study phenomenon were then named with phrases or statements that concisely portray the embedded notions within the themes. Eventually, extracts from the narratives of participants were selected to serve as examples to demonstrate the essence of each theme, establishing a plausible and relevant account of the phenomenon under study.

### Trustworthiness

The trustworthiness of the present study was maintained by establishing four major criteria, namely, credibility, transferability, dependability, and confirmability (Lincoln & Guba, 1985). To address credibility, member checks were performed to validate the findings with the participants so as to ensure their thoughts were appropriately captured in the data. Transferability was achieved through the provision of thick descriptions of the findings and research process, which enables future evaluations of whether the findings could be extended to other possible contexts. For dependability, it was established by implementing an inquiry audit to allow scrutiny of the appropriateness of the research steps, as well as the sincerity of the findings. Confirmability was addressed by maintaining a reflexive journal to record and substantiate the research process, notably the development of themes from the data.

## Results

The findings of the present study illustrate five overarching themes that describe emergency nurses’ perception of risk appraisal regarding the EID situations in the emergency care context. These themes are (1) the novelty of an EID, (2) the severity of an EID, (3) the proximity to an EID, (4) the complexity of an EID situation, and (5) the response levels towards an EID situation.

### The novelty of an EID

The participants revealed that their appraisal of the situated circumstance would incorporate their individual understanding of a particular EID scenario. For example, participants suggested the novelty of an EID could affect their appraisal of a disease situation. Although the term “emerging infectious disease” has been coined to signify that this disease group has newly appeared, emergency nurses’ familiarity with an epidemic event could vary from disease to disease. One participant referred to her personal experience in the management of the Ebola virus disease during the epidemic event’s global outset as follows:

“The Ebola virus disease is new to us, and we do not know much about the disease, we cannot predict how much worse the disease situation will be, we do not know how high the risk is that we will be infected. Not long ago, there was a local outbreak of H7N9 avian influenza. It was also regarded as an emerging infectious disease, but there had not been much reaction from our staff. Perhaps it is because we knew that the avian influenza was limited to poultry-to-human transmission, and it was easier for us to identify suspected cases and take the necessary precautions.” (P18)

From the remarks of the above participant, it is revealed that the Ebola virus disease outbreak was regarded as a novel challenge to emergency nurses because of a lack of understanding and clarity on the nature of the disease and the severity of the threat it posed. Indeed, this view was echoed by another participant, who recalled the unprecedented hardships from emergency nurses’ participation in the management of the severe acute respiratory syndrome (SARS) pandemic, while facing the threat from Ebola virus disease:

“Those new infectious diseases (EIDs) have been unsettling for us. The Ebola virus disease is a typical example. We have no idea what we can do for patients. And we do not know what we can do for ourselves … we did not know what we were supposed to do, we did not know whether we were protected from infection.” (P17)

This reveals that the novelty of an EID could influence emergency nurses’ understanding and experience of their participation in an epidemic event. Study participants indicated their difficulties in coping with EIDs with which they were unfamiliar. Concerns were also expressed about the risk of handling a new infectious disease while nurses only had limited knowledge of the nature of the disease, including the mode of transmission and the consequences of infection.

### The severity of an EID

Another important consideration for participants in their appraisal of EID disease situations is associated with the severity of the disease. The participants’ understanding of the severity of an epidemic was mainly determined by their impressions of the impact of the disease on the public. The majority of participants stated that the foremost position of the ED in healthcare service provision allowed them to recognize the havoc caused by EIDs in the community, and therefore they were able to realize and appraise the disease situation in their everyday work. One of the major elements that determine the severity of an EID and its impact on the general public was associated with the lethality of the disease, which, in other words, was the deadliness of the disease in terms of the human death toll. A nursing officer who had experienced the immense hardships at the time that SARS wreaked havoc around the globe remarked that some emergency nurses fled in panic during the outbreak, because of the high mortality rates among infected patients. As he said:

“At the time of SARS, some of our colleagues requested to be reallocated to other departments instead of staying in the ED, while the disease had triggered the resignation of some nurses in our department. They thought the SARS was deadly because the mortality rate from infection is high. Therefore, they wanted to leave, as they perceived that the disease situation had become perilous and deplorable.” (P6)

In addition to an EID’s fatal consequences among the human population, the participants also highlighted that in appraising the severity of the disease situation, they would take into consideration the infectiousness of the disease. According to the participants, the infectiousness of disease was characterized by the scale of the spread of disease with reference to the magnitude of infections in the population. In their accounts of disease transmissibility, the majority of participants agreed that an EID’s disease situation would be regarded as serious if there was a widespread outbreak of the disease that infected a large number of people. A nursing officer indicated how she made sense of the transmission route of different EIDs while understanding their infectiousness as follows:

“The mode of transmission is an important part of the nature of a disease that one has to understand in order to deal with it. For example, if I am to compare Ebola with SARS, in my personal understanding, the latter will be more devastating. It is because SARS could spread through an airborne route, while Ebola transmission is restricted to direct contact.” (P16)

Regarding the participants’ narratives, the severity of an EID was not determined by either mortality or incidence figures alone. Instead, the combination of both its deadliness and infectiousness were considered by emergency nurses when evaluating an EID situation. A participant offered an explanation for the uniqueness of SARS, stated that it was a devastating EID because it demonstrated both high lethality and transmissibility:

“For swine flu (H1N1), it spread very fast among the population, but it was usually not lethal, it was just like the common flu with the usual mortality rate. For avian flu (H7N9), it was deadly, but the risk of infection is relatively low, as the probability of human-to-human infection has remained minimal. However, SARS was different. It was both a deadly and highly infectious disease.” (P6)

Apparently, the severity of an EID was regarded as important for emergency nurses in appraising the risk from a disease scenario they were facing. It was revealed that both the deadliness and the infectiousness of disease were crucial for individuals to acknowledge and recognize a disease situation.

### The proximity to an EID

It was also suggested by the majority of participants that the proximity to an EID would influence their understanding of the disease situation. Participants referred to this feature as the geographical nearness of the impact of an epidemic event. There were some suggestions that proximity to an EID outbreak would influence their understanding of the seriousness of a disease situation, which sometimes superseded the mortality and morbidity of an EID. For instance, a nursing officer, who was interviewed at the time the Ebola virus disease outbreak was confined to the West Africa region, expressed her view that the threat from the disease was reasonable without inducing apprehension, because of the considerable geographical distance of the outbreak from their region:

“Although the Ebola virus disease is a deadly disease with a high mortality rate, it is still far away from us as there are fortunately no reported cases in Hong Kong. The disease is limited to West Africa, which is far away from us. We will still remain vigilant in the future and need not panic about the disease situation at this moment.” (P16)

This view was echoed by another participant, who commented on the scenario of the Middle East respiratory syndrome (MERS) as follows:

“I think the infection control precautions for MERS should be stepped down because the outbreak has long been limited to countries of the Middle East. There have only been sporadic cases.” (P1)

Interestingly, another participant expressed her views on the MERS epidemic in the opposite manner, after the epidemic struck South Korea and spread in close proximity to Hong Kong:

“We have to pay special attention and be extra cautious about MERS because it is coming near us and could hit us at any time.” (P19)

From the information provided by the participants, it is recognized that their understanding of the risk from an EID event could vary, depending on the disease’s impact from a geographical perspective.

### The complexity of an EID situation

Together with the novelty, severity, and proximity of an EID, participants also underlined that complexity was a significant feature in appraising the risk from an epidemic situation. From the participants’ perspectives, an EID situation becomes complex if multiple diseases have emerged and have been brewing simultaneously. There were some suggestions that the EID situation had been complex all along since multiple EIDs have continually coexisted in the human population. As one participant put it:

“EIDs have always been here around us. They are always here. It is just the trend of the infection that differs from time to time; that is, a strain of EID is dominant at this time, then another strain will take its place and become dominant later.” (P15)

Even worse is what was reported by participants as a common situation in their daily work, which was the dominance of multiple EIDs concurrently. The comment below illustrates the complexity of the circumstance that emergency nurses encountered, with the presence of three different strains of EID. The participant used a mahjong metaphor to describe the disease situation as follows:

“At this particular moment, an outbreak of a large-scale infectious disease is already ‘ready hands’ (on the verge of breaking out), and waiting for three tiles (triggers). In front of us, there are the Ebola virus disease, Middle East respiratory syndrome, and H7N9 avian influenza waiting to strike.” (P24)

Participants’ narratives clearly identify the fact that the risk perceived by emergency nurses during their involvement in disease management was comprised of intertwined scenarios of various EIDs. Such scenarios were regarded by the participants as the constituents of the complexity in appraising the risk from an EID situation.

### Response levels towards an EID situation

Other than the actual nature and characteristics of an EID situation, another major consideration for the participants in appraising the risk of the disease situation originated from the response level of the government response system. This system, which envisages a three-tiered response level (Alert, Serious, and Emergency), is a part of the preparedness plan announced by the Hong Kong government to depict epidemiological scenarios. The response levels are established based on the assessment of the outbreak risk of an EID and its impact on human health and healthcare organizations. The majority of participants commented that the response level for an EID is the most influential aspect in defining a disease situation. For example, one participant described how the progress of the EID disease situation could alter their practice as follows:

“Whenever there are changes in the alert level, for example, the level has been stepped up from Alert to Serious this month, there have been some corresponding modifications to our practice in workflow and disease prevention. I would then realise the situation is getting worse, and I am in greater danger.” (P4)

The above narrative illustrates that the response level is an important component when emergency nurses are appraising the risk surrounding the situations they encounter during EID management.

## Discussion

The findings of the present study have explored emergency nurses’ perceptions regarding the risks appraisal of the threat of the EID situation in the emergency department context. Taken together, these findings suggest that emergency nurses take an array of factors into account in their appraisal of an EID disease situation. The participants mentioned that their risk perceptions would be grounded in their impressions and interpretations of the infectiousness, severity, proximity, and complexity of an EID situation. Meanwhile, the response level was also considered by the participants as a prominent determinant for emergency nurses in their appraisal of threats from an EID situation. The findings have offered a new perspective on examining the impacts of EID on emergency nurses, addressing the existing knowledge gap in understanding their risk appraisal over the course of an epidemic.

Most of the participants indicated that the vulnerability to infection from an EID is of their primary concern for risk appraisal. According to the participants’ description, such a vulnerability is portrayed as the novelty, severity, proximity, and complexity of an EID and its corresponding situation. In line with those previous studies, the risk of exposure to a disease is often considered an important factor that influences nurses’ risk perception. Findings from various studies have identified an elevated infection rate in nurses during EID events. For example, a small-scale study by Wise et al. (Wise et al., 2011) reported that nurses were significantly susceptible to influenza infection during the H1N1 pandemic in 2009. In addition, in a previous study (Olu et al., 2015) of Ebola virus disease (EVD) transmission in hospital workers, it was identified that over half of the infections occurred in nurses, despite the fact that they had previously been trained in infection prevention. Indeed, nurses considered physical exposure to patients with confirmed or probable infection to be the significant risk factor in the course of their work (Cheong et al., 2007; Imai et al., 2008; Tzeng & Yin, 2006; Wong et al., 2008). A possible explanation for the perceived risk of occupationally acquired infection among nurses was that nurses are frequently engaged in high-risk patient care activities during routine clinical practice. These patient care activities, such as suctioning, nasogastric feeding, and intubation of infected patients, can put nurses at risk for infection. In addition, nurses’ practice settings are considered to significantly affect their perceived occupational risk of infection. Emergency nurses were frequently cited to demonstrate additional perceived risks for contracting an infection (Parmeggiani, Abbate, Marinelli, & Angelillo, 2010). It is also reported that emergency nurses found themselves particularly vulnerable and susceptible to infections during an EID outbreak (Lam & Hung, 2013; Venkat et al., 2012). It is suggested in the literature that emergency nurses’ risk perceptions were associated with the additional threat embedded in the practice of emergency care, in which they constitute the public health frontline of defence in the course of an outbreak. Indeed, the environment of EDs is commonly considered a high-risk workplace, where emergency nurses are prone to outbreak exposure (Wu et al., 2009).

Another important finding of the present study is the influences of the response level for an EID on emergency nurses’ risk appraisal of EID situations. It is underlined in the findings that the existing index and scale classifying the severity of EIDs are important indicators for emergency nurses to address the disease situations in epidemic events. Indeed, the response level clearly signposts the progress of an EID and strongly influences emergency care delivery and emergency nurses’ routine practice. However, the current international classification scheme, namely the World Health Organization (WHO) global phase of pandemic alert (World Health Organization, 2009), only reflects the severity and progress seen in an influenza pandemic. There is an absence of a consensual categorization system for other EIDs, which has resulted in uncertainty and discrepancies in assessing the risk and seriousness of non-influenza EID events. A key policy priority should, therefore, be to devise a well-established alert system, which could accommodate disease situations for various EIDs. Such an alert system could offer essential information regarding the evolving picture of different EID events at the local, national, and global level, providing guidance to healthcare facilities and institutes in preparing a response to EIDs. Frontline healthcare workers, especially emergency nurses, could then appraise a disease situation with improved clarity, which might reduce their uncertainty, contributing to improved alertness and vigilance towards possible EID outbreaks.

Although the primary focus of the present study was on the factors associating with emergency nurses’ risk appraisal of an EID, the influence of perceived risk on emergency nurses’ practice merits attention. As portrayed in the findings, emergency nurses developed different levels of a sense of anticipated crises in the face of different EID events, which might create considerable fear and anxiety as to their personal wellness. Interestingly, it is suggested in the literature that such a sense of crisis might foster nurses’ awareness of the severity of the disease situation, enhancing their vigilance against EIDs. On the other hand, it has previously been observed that if healthcare workers underestimate the severity of a public health event, it could result in laxness in handling and fulfiling the tasks required (Balicer, Omer, Barnett, & Everly, 2006; Cava, Fay, Beanlands, McCay, & Wignall, 2005). Thus, the findings shed some light on the importance of maintaining an optimal level of risk perception and crisis awareness among nurses during an EID event, to promote their alertness and prudence at work but without triggering panic.

## Limitations

The scope of this study was limited in several ways. The major limitation of the present study is that the risk appraisal of emergency nurses can only be considered as one of the factors that would affect emergency nurses’ behaviours in the course of EID management. In addition, the nature and characteristics of an EID situation might not exclusively constitute the participants’ risk appraisal. Thus, further investigations incorporating other possible factors, such as cultural issues or organizational environment, could be considered. Notwithstanding these limitations, the present study offered useful insights into the understanding of the factors influencing the risk appraisal towards EIDs among emergency nurses.

## Conclusions

This study set out with the aim of exploring the important nature of an EID situation that influences the risk appraisal among emergency nurses from their perspectives. This is the first study that focused on emergency nurses’ perceptions on their appraisal regarding the threats from an EID event. The findings have delineated the several factors that might contribute to emergency nurses’ attitudes and practices in the course of an EID event, which included the novelty of an EID, the severity of an EID, the proximity to an EID, the complexity of an EID situation, and the response levels towards an EID situation. These findings offer valuable information in understanding the nature of an EID that is related to the emergency nurses’ perceived risk. It is anticipated that the information may help to predict the attitudes and behaviours of emergency nurses in future impending epidemic events, enhancing emergency nurses’ preparedness in such situations.

## Data Availability

To access data for further analysis, please contact the Corresponding Author via stanleylam@twc.edu.hk.
